# Geospatial clustering reveals dengue hotspots across Brazilian municipalities, 2024

**DOI:** 10.3389/fpubh.2025.1620914

**Published:** 2025-10-27

**Authors:** Brena F. Sena, Bobby Brooke Herrera, Danyelly Bruneska Gondim Martins, Jose Luiz Lima Filho

**Affiliations:** ^1^Keizo Asami Institute, Universidade Federal de Pernambuco, Recife, Brazil; ^2^Rutgers Robert Wood Johnson Medical School, Department of Medicine, Division of Allergy, Immunology, and Infectious Diseases, and Child Health Institute of New Jersey, Rutgers University, New Brunswick, NJ, United States; ^3^Rutgers University, Rutgers Global Health Institute, New Brunswick, NJ, United States; ^4^Department of Biochemistry, Universidade Federal de Pernambuco, Recife, Brazil

**Keywords:** dengue, spatial epidemiology, DBSCAN, clustering, Brazil, hospitalization, rainfall, public health surveillance

## Abstract

**Introduction:**

Dengue virus (DENV) remains a major and recurrent public health challenge in Brazil. In 2024, the country experienced its largest recorded epidemic, with more than six million probable cases and substantial pressure on hospital systems. The epidemic’s highly heterogeneous burden highlights the need for municipal-scale geospatial analyses to identify actionable hotspots for targeted interventions.

**Methods:**

We conducted a nationwide clustering analysis using dengue case notifications and hospitalizations from the national SINAN surveillance system, with denominator populations from the Brazilian Institute of Geography and Statistics (IBGE). We calculated standardized case and hospitalization rates per 100,000 population for all municipalities. A multivariate density-based spatial clustering algorithm (DBSCAN) integrated municipality centroids with epidemiologic burden. Parameters (eps, minPts) were selected using k-distance inspection and sensitivity analyses. Temporal stability was assessed through monthly DBSCAN runs using a common parameter set, and climatic associations were evaluated by pairing dengue indicators with CHIRPS precipitation at 0–3 monthly lags.

**Results:**

DBSCAN identified 25 high-burden municipal clusters, with 5,111 municipalities (92.6%) clustered and 408 (7.4%) were classified as noise. Several clusters exhibited average case rates exceeding 20,000 per 100,000 population, particularly in Minas Gerais, Paraná, and Bahia. Some high-incidence municipalities remained geographically isolated and unclustered. Hospitalization-only clustering produced similar geographic patterns. Monthly analyses revealed persistent high-burden clusters, and precipitation was positively associated with incidence at an approximately two-month lag.

**Discussion:**

This study demonstrates that integrating spatial, temporal, and climatic dimensions into a DBSCAN framework provides a reproducible method for delineating dengue hotspots at the municipal scale. By distinguising high-intensity clusters from low-burden areas, the approach offers and operationally relevant tool for guiding vector control and outbreak response during dengue epidemics in Brazil.

## Introduction

Dengue virus (DENV) remains one of the most pressing vector-borne public health threats in Brazil, causing recurrent epidemics that vary widely in magnitude and geography. Four antigenically distinct serotypes (DENV-1-4) co-circulate in Brazil with regional and temporal variation. Primary infection typically confers lifelong immunity to the infecting serotype but transient cross-protection to others; subsequent heterotypic infections elevate the risk of severe outcomes via antibody-dependent enhancement (1–3). The 2024 season marked Brazil’s largest recorded dengue epidemic, with more than six million probable cases and tens of thousands of hospitalizations, imposing substantial strain on health systems (4). Because epidemic intensity varies over short distances, national and even state summaries can obscure actionable hotspots relevant to vector control, clinical surge planning, and targeted communication.

During epidemic peaks, routine laboratory confirmation is limited and most SINAN notifications rely on clinical-epidemiologic criteria (5). In this operational reality, geospatial methods that use syndromic notifications and hospitalizations can identify places where transmission is unusually intense or persistent even when virologic typing is incomplete. Spatial epidemiology offers multiple approaches, but several common methods require strong assumptions: k-means demands a pre-specified number of clusters and favors spherical geometries, and scan statistics impose moving windows that may not align with municipal boundaries. Density-Based Clustering of Applications with Noise (DBSCAN) is attractive because it does not require pre-specifying the number of clusters, can recover irregular shapes, and explicitly labels “noise,” separating isolated outliers from coherent high-density zones (6, 7). Although DBSCAN has seen growing use in infectious-disease surveillance internationally (8, 9), national-scale applications at Brazil’s municipal resolution remain limited. We implement DBSCAN on a joint feature space combining municipal centroids with standardized case and hospitalization rates.

Here we present a national application of multivariate DBSCAN framework that integrates municipal geography (centroids) with standardized epidemiologic burden (case and hospitalization rate per 100,000) to delineate dengue hotspots in 2024. We selected parameters (eps, minPts) using k-distance diagnostics and sensitivity checks (Supplementary Figure S1; Supplementary Table S1). Because response planning requires both spatial and temporal perspectives, we reran DBSCAN monthly using a common parameter set to assess persistence, the extent to which municipalities and populations remain in clusters across consecutive months (Supplementary Figure S2; Supplementary Table S4). Given the established linkage between rainfall and *Aedes aegypti* dynamics, we paired municipal dengue indicators with CHIRPS precipitation at 0–3 lags to characterize short-lag climate associations and operational lead time (27, 28).

**Figure 1:**
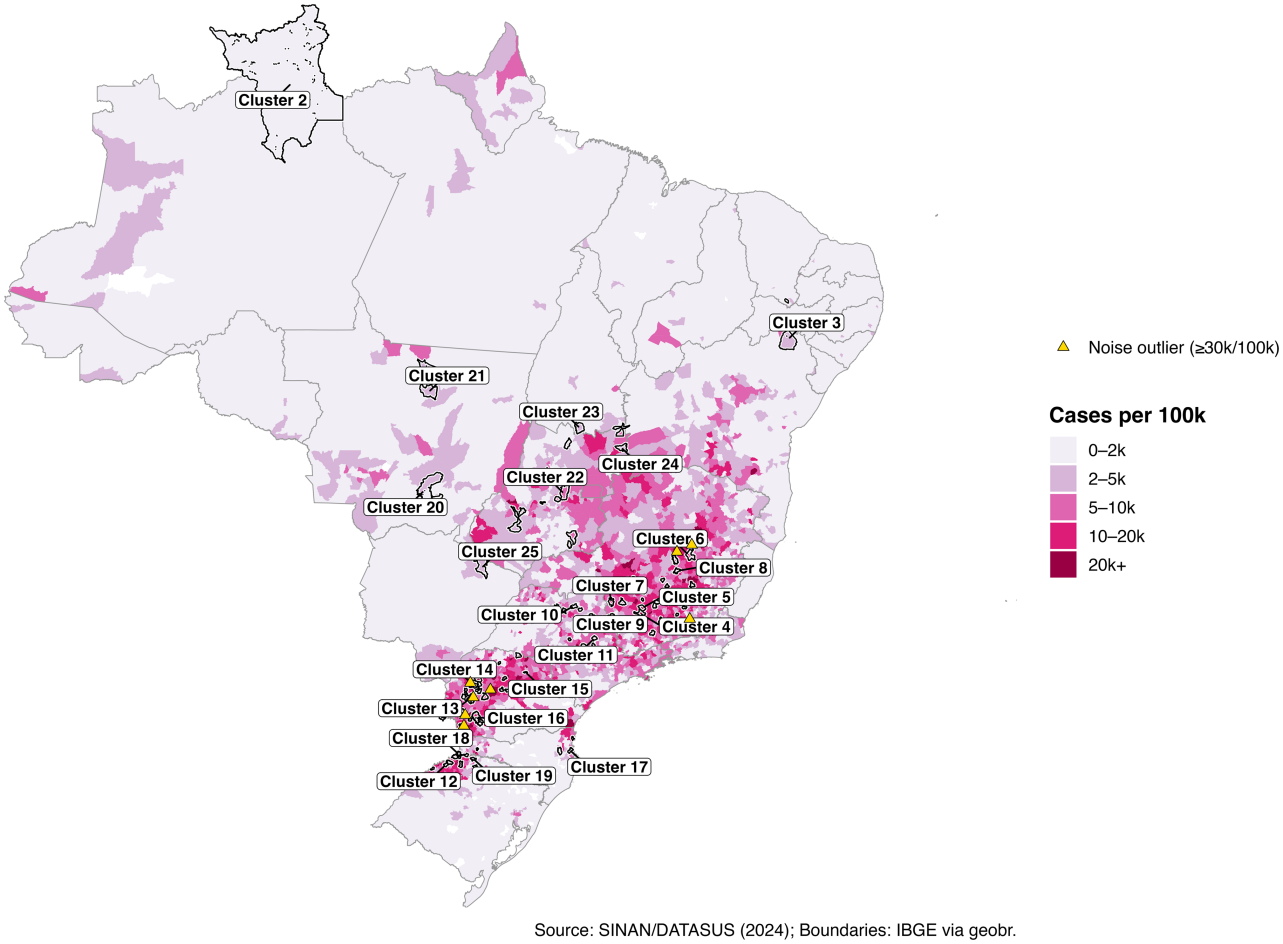
National map of dengue case rates and DBSCAN multivariate clusters (Annual, 2024).

**Figure 2:**
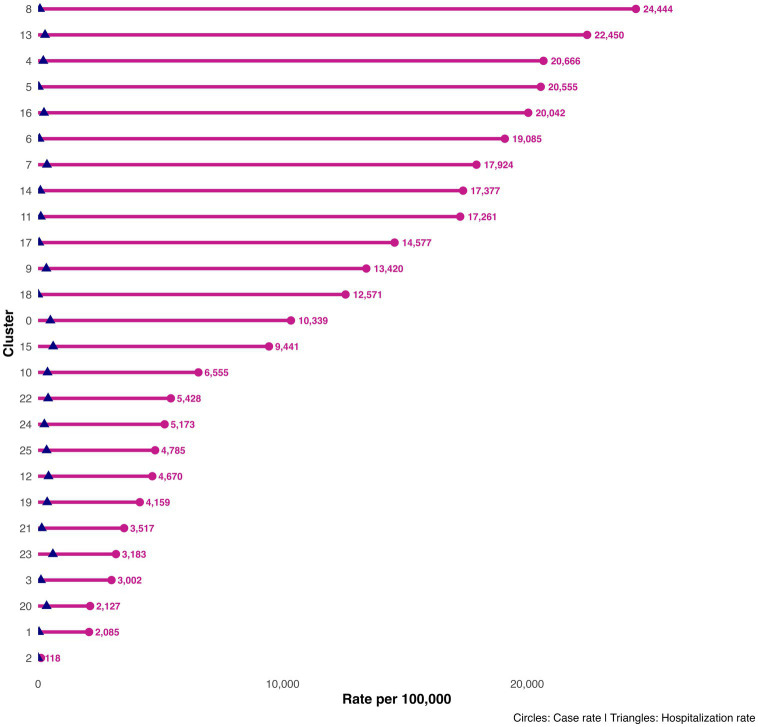
Cluster-wise averages (cases and hospitalizations per 100 k) (2024).

**Figure 3:**
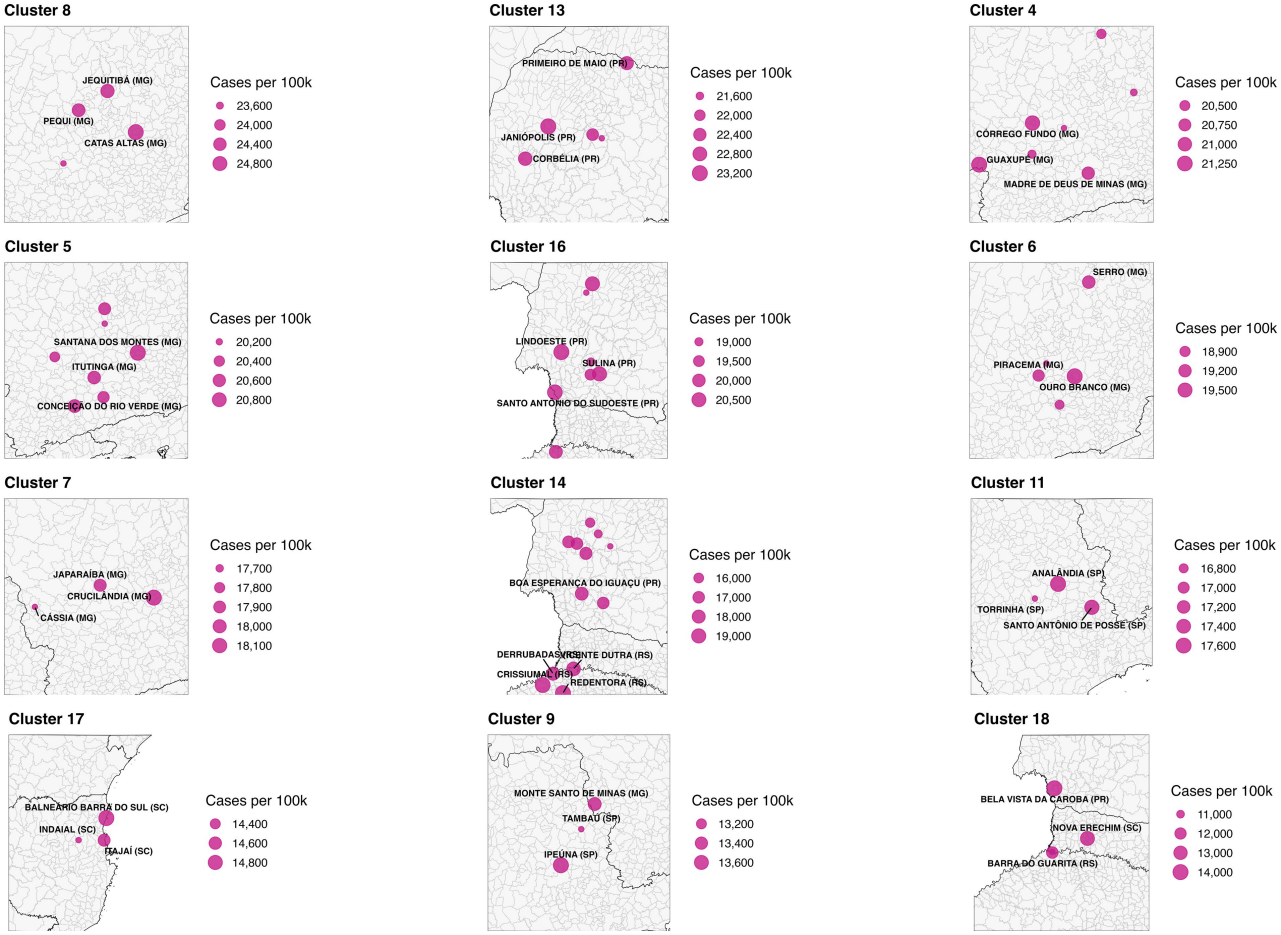
Composite zoom of selected high-burden dengue clusters. Clusters were identified using DBSCAN spatial clustering based on the geographic coordinates (centroids) of Brazilian municipalities. To prioritize areas of highest public health relevance, only spatial clusters with elevated average dengue case rates and hospitalization rates per 100,000 population were included. Each panel represents a distinct high-burden cluster. Municipalities within the cluster are shown as pink circles, scaled by dengue case rate. The three municipalities with the highest per capita case rates in each cluster are labeled. State boundaries are overlaid for geographic reference. Source: SINAN/DATASUS (2024); Shapefiles from IBGE via the geobr R package.

**Figure 4:**
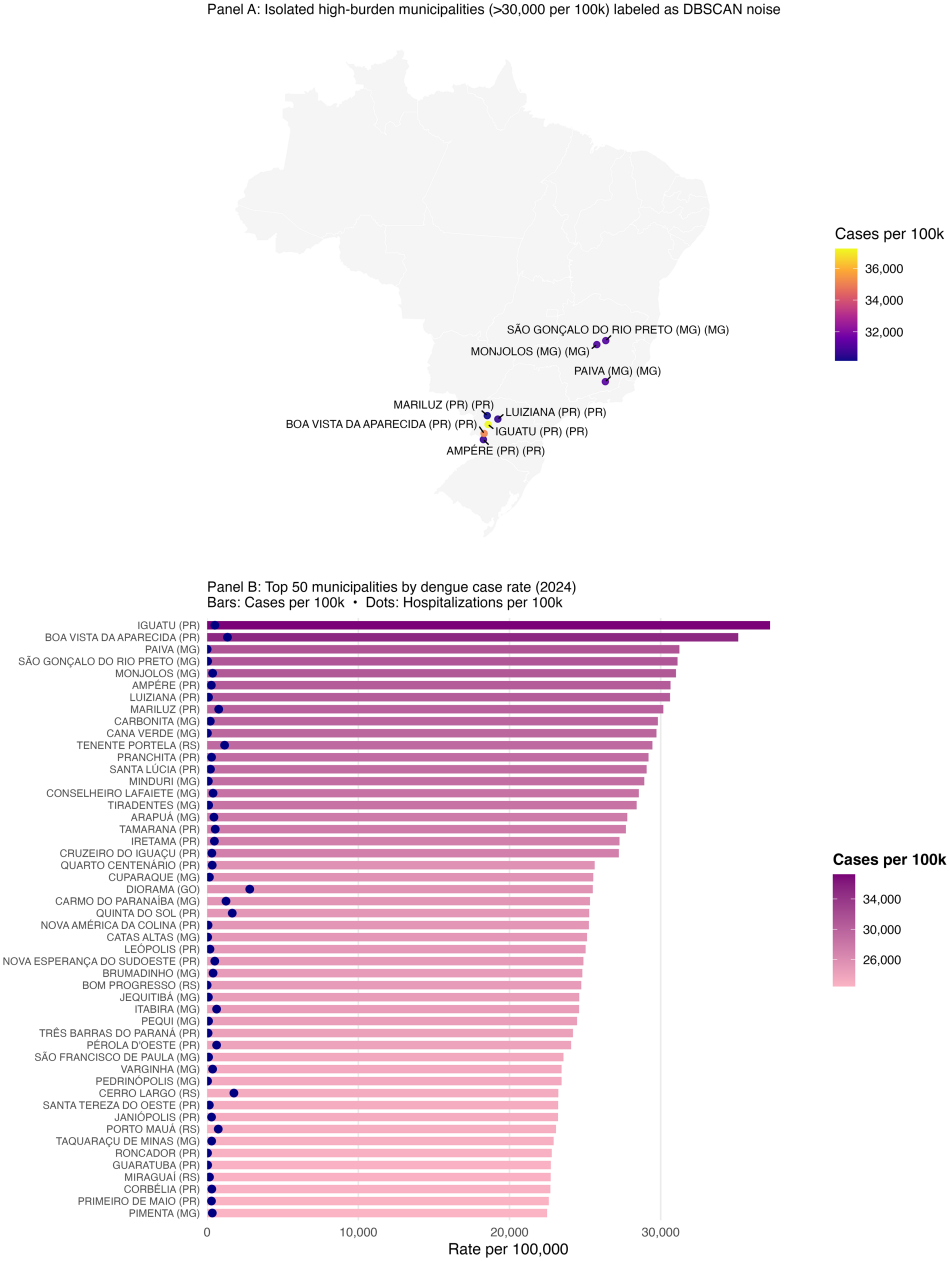
Outlier municipalities with extreme dengue burden and the top 50 municipalities by case rate (2024). Geographically isolated municipalities with >30,000 dengue cases per 100,000 that DBSCAN labeled as noise are shown explicitly; these outliers remain epidemiologically important despite algorithmic exclusion in clustering. Top municipalities by annual case rate (2024), ranked; full list in Supplementary Table S3.

**Figure 5:**
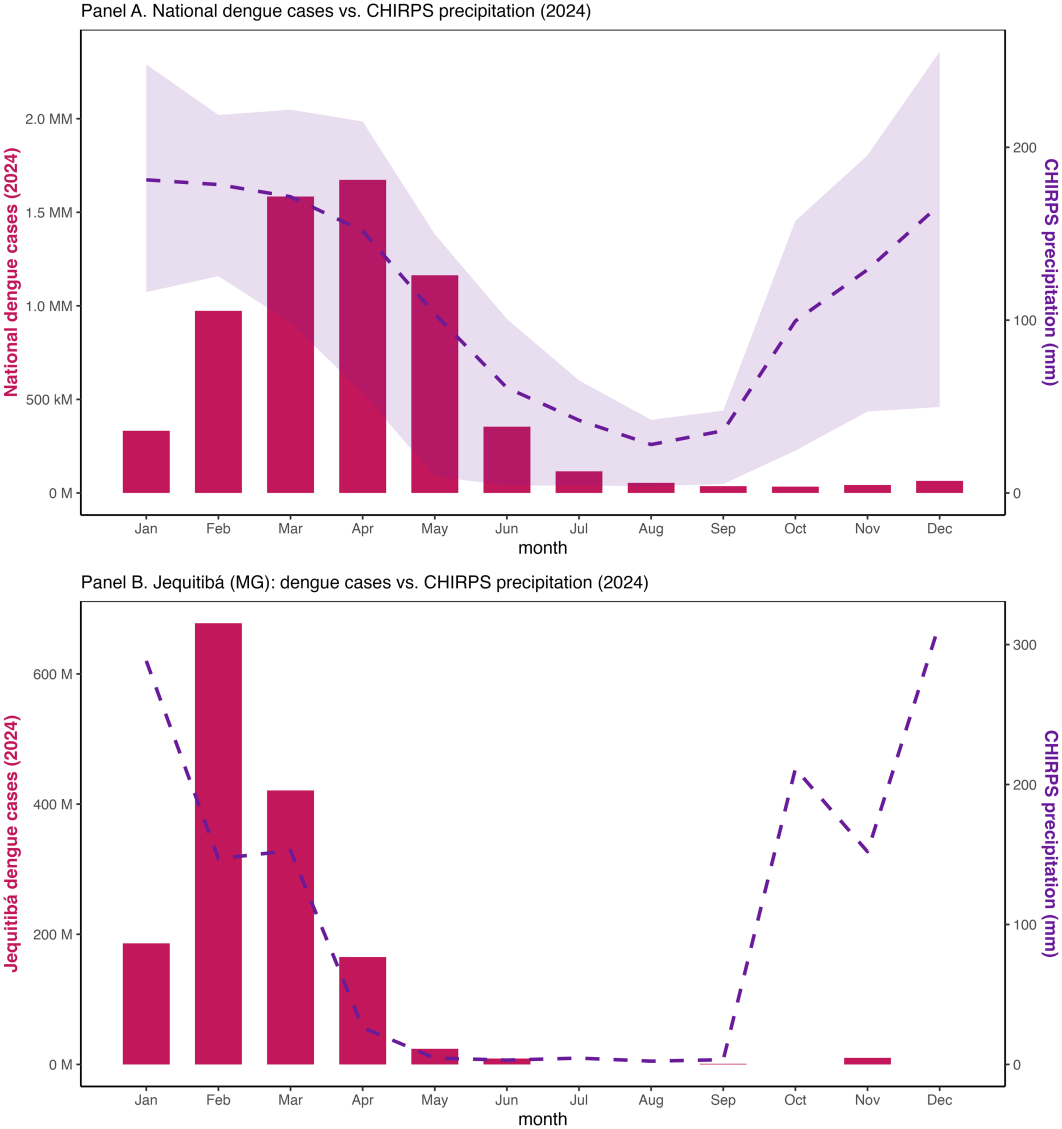
National monthly precipitation (CHIRPS) with dengue totals and at Jequitibá, Minas Gerais (2024).

We address three practical questions: (i) Where are the municipal clusters of greatest burden when geography and epidemiology jointly determine membership? (ii) How stable are these patterns over the epidemic year? (iii) How do short-lag rainfall patterns relate to the observed spatial structure? To aid interpretation, we treat “Cluster 1” as a low-burden background, focus on higher-intensity clusters (ID >1), and preserve isolated high-incidence municipalities labeled as noise (ID = 0). The resulting products include a national map of multivariate clusters (Figure 1); cluster-level summaries (Tables 1, 2; Figure 2); zoomed composite for exemplar high-burden areas (Figure 3); an outlier panel that preserves isolated hotspots (Figure 4); a climate-aligned national panel (Figure 5); and diagnostics and robustness checks (Supplementary Figures S1–S4; Supplementary Tables S1–S5).

**Table 1:** Summary of DBSCAN-identified clusters.

Cluster	No. of municipalities	Total cases	Total hospitalizations	Avg. cases per 100 k	Avg. hospitalizations Per 100 k
8	4	5,450	17	24,444	76.6
13	5	8,996	117	22,450	284.2
4	7	27,584	269	20,666	212.3
5	7	12,726	18	20,555	27.0
16	8	19,103	220	20,042	240.4
6	5	16,625	58	19,085	54.0
7	3	4,954	98	17,924	361.1
14	12	20,869	102	17,377	93.8
11	3	6,563	41	17,261	111.4
17	3	55,144	286	14,577	47.0
9	3	6,660	168	13,421	337.6
18	4	3,544	3	12,571	4.4
15	3	1,373	89	9,441	616.5
10	8	92,927	5,086	6,555	384.9
22	5	8,054	638	5,428	409.4
24	3	1,505	75	5,173	256.0
25	3	2,339	177	4,785	348.6
12	6	17,345	1,447	4,670	425.7
19	3	949	84	4,159	370.9
21	3	835	39	3,517	150.1
23	3	958	208	3,183	604.8
3	3	1,045	43	3,002	117.0
20	3	2,088	301	2,127	347.5
1	4,984	4,530,651	110,091	2,085	37.7
2	15	624	28	118	4.3

**Table 2:** Municipality composition of high-burden clusters.

Cluster	No. of municipalities	Municipalities
8	4	Catas Altas (MG), Jequitibá (MG), Pequi (MG), São Francisco De Paula (MG)
13	5	Arapuã (PR), Ariranha Do Ivaí (PR), Corbélia (PR), Janiópolis (PR), Primeiro De Maio (PR)
4	7	Boa Esperança (MG), Carmo Da Mata (MG), Córrego Fundo (MG), Guaxupé (MG), Madre De Deus De Minas (MG), Presidente Juscelino (MG), São Gonçalo Do Rio Abaixo (MG)
5	7	Conceição Do Rio Verde (MG), Cristais (MG), Florestal (MG), Itatiaiuçu (MG), Itutinga (MG), Santana Dos Montes (MG), São Vicente De Minas (MG)
16	8	Lindoeste (PR), Moreira Sales (PR), Quedas Do Iguaçu (PR), Santo Antônio Do Sudoeste (PR), Sulina (PR), São Jorge D’Oeste (PR), Tuneiras Do Oeste (PR), Vista Gaúcha (RS)
6	5	Dores De Campos (MG), Ouro Branco (MG), Piracema (MG), Rio Manso (MG), Serro (MG)
7	3	Crucilândia (MG), Cássia (MG), Japaraíba (MG)
14	12	Boa Esperança Do Iguaçu (PR), Braganey (PR), Cafelândia (PR), Crissiumal (RS), Derrubadas (RS), Goioerê (PR), Juranda (PR), Nova Cantu (PR), Redentora (RS), São João (PR), Tupãssi (PR), Vicente Dutra (RS)
11	3	Analândia (SP), Santo Antônio De Posse (SP), Torrinha (SP)
17	3	Balneário Barra Do Sul (SC), Indaial (SC), Itajaí (SC)
9	3	Ipeúna (SP), Monte Santo De Minas (MG), Tambaú (SP)
18	4	Barra Do Guarita (RS), Bela Vista Da Caroba (PR), Itapiranga (SC), Nova Erechim (SC)
15	3	Kaloré (PR), Nova Santa Bárbara (PR), Rosário Do Ivaí (PR)
10	8	Boracéia (SP), Colina (SP), Ribeirão Preto (SP), Santa Rosa Da Serra (MG), São José Do Rio Preto (SP), São João Batista Do Glória (MG), Tabapuã (SP), Viradouro (SP)
22	5	Americano Do Brasil (GO), Caldas Novas (GO), Petrolina De Goiás (GO), Pirenópolis (GO), Água Limpa (GO)
24	3	Campos Belos (GO), Divinópolis De Goiás (GO), Guarani De Goiás (GO)
25	3	Chapadão Do Sul (MS), Ivolândia (GO), Montividiu (GO)
12	6	Capitão Leônidas Marques (PR), Foz Do Iguaçu (PR), Horizontina (RS), Jesuítas (PR), Presidente Castelo Branco (PR), Ângulo (PR)
19	3	Constantina (RS), Planalto (RS), Porto Vera Cruz (RS)
21	3	Cláudia (MT), Nova Guarita (MT), Terra Nova Do Norte (MT)
23	3	Formoso (GO), Itapaci (GO), Palmeirópolis (TO)
3	3	Chorrochó (BA), Macururé (BA), Porteiras (CE)
20	3	Campo Verde (MT), Jaciara (MT), Juscimeira (MT)
1	4,984	N/A
2	15	Alto Alegre (RR), Amajari (RR), Boa Vista (RR), Bonfim (RR), Cantá (RR), Caracaraí (RR), Caroebe (RR), Iracema (RR), Mucajaí (RR), Normandia (RR), Pacaraima (RR), Rorainópolis (RR), São João Da Baliza (RR), São Luiz (RR), Uiramutã (RR)

## Methods

### Data sources and outcome measures

We conducted a nationwide analysis of dengue case notifications and hospitalizations reported in 2024, using the publicly available SINAN (Sistema de Informação de Agravos de Notificação) database maintained by Brazil’s Ministry of Health (5). Population estimates for all 5,570 municipalities were obtained from the Brazilian Institute of Geography and Statistics (IBGE) (25) to calculate standardized burden measures. Two primary outcomes were defined for each municipality: (i) dengue case notifications per 100,000 population and (ii) dengue-related hospitalizations per 100,000 population. Municipalities with missing population or outcome data were excluded.

### Climate data integration

To ensure uniform national coverage, monthly total precipitation for 2024 was obtained from CHIRPS (Climate Hazard Group InfraRed Precipitation with Station data; ~5 km spatial resolution) (37, 38). Preliminary checks with station data from INMET (26) confirmed consistency but were not used in final analyses. For each municipality, precipitation was computed by averaging all CHIRPS grid cells intersecting its polygon. We constructed monthly lags of 0–3 months to evaluate rainfall-dengue associations at macroregional scale and to visualize national seasonality alongside national dengue totals (Figure 5). Local station data explored in preliminary work were not required for the final analyses presented here; we retained CHIRPS to maintain consistency and coverage.

### Spatial clustering analysis

We applied DBSCAN (Density-Based Spatial Clustering of Applications with Noise) to identify spatially contiguous clusters of elevated burden without imposing assumptions about cluster number or geometry (6, 7).

Inputs included municipal centroid coordinates (latitude, longitude) and standardized burden measures (z-scores of case and hospitalization rates). We examined k-distance plots to inform the neighborhood radius (eps) and minPts values (Supplementary Figure S1; Supplementary Table S1). For each cluster, we computed the number of municipalities, total cases and hospitalizations, and mean burden levels (Table 1).

### Temporal clustering and persistence

To assess temporal stability, we ran DBSCAN on monthly municipal data using the same parameter values. For each month, we summarized the number of clusters, the proportion of municipalities assigned to any cluster, and the share of the national population located in municipalities classified as cluster members. We identified municipalities that were cluster members for ≥3 consecutive months and calculated the size of this persistent cluster population (Supplementary Table S4). Monthly maps depict cluster membership as magenta points over a national basemap (Supplementary Figure S2).

### Spatial autocorrelation

We computed Global Moran’s I for municipality case rates using queen contiguity and a row-standardized spatial weight matrix (spdep). We reported Moran’s I, its standard deviate, and *p*-value under randomization (Supplementary Table S2), with a Moran scatterplot provided for illustration (Supplementary Figure S3).

### Socioeconomic comparisons

To explore socioeconomic context, we paired DBSCAN cluster status with 2020 municipal GDP per capita from IBGE (14). We compared distributions between clustered and non-clustered municipalities and fit a logistic regression with cluster membership as the dependent variable and GDP per capita as the independent variable, recognizing that this cross-sectional approach captures association rather than causation.

### Statistical environment and visualization

All analyses were conducted in R (version 2024.12.1+563) using sf, geobr, dbscan, spdep, dplyr, tidyr, ggplot2, and related packages. Figures 1–5 compose the main results; Supplementary Figures S1–S4 and Supplementary Tables S1–S5 contain diagnostics, sensitivity analyses, and supporting materials.

## Results

Using a multivariate DBSCAN with eps = 0.3 and minPts = 3, we identified 25 geographically coherent clusters along with a large low-burden background (Cluster 1) and a small northern regional cluster (Cluster 2). In total, 5,111 municipalities (92.6%) were assigned to a cluster and 408 (7.4%) labeled as noise. Global Moran’s I for municipal case rates was strongly positive (I = 0.598; z = 72.8; *p* < 0.001), confirming non-random spatial autocorrelation consistent with the clustering patterns (Supplementary Table S2; Supplementary Figure S3).

High-burden clusters were geographically concentrated and often compact. Minas Gerais contained several of the most intense clusters, including a four-municipality cluster (Cluster 8: Jequitibá, Pequi, São Francisco de Paula, and Catas Altas) with average case rates of 24,000 per 100,000 and hospitalization rates of 76.6 per 100,000. Similarly, in Paraná, Cluster 13 (Arapuã, Ariranha do Ivaí, Corbélia, Janiópolis, and Primeiro de Maio) presented average hospitalization burdens of 284.2 per 100,000, while other southern clusters (e.g., Cluster 15) reached hospitalization burdens above 600 per 100,000. Cluster 4, concentrated in southern Minas Gerais and containing municipalities such as Guaxupé and São Gonçalo do Rio Abaixo, also demonstrated high overall burden (Table 3). These clusters are visualized at the national scale (Figure 1), with zoomed-in composite maps (Figure 3) and full municipal composition listed in Table 2, 3.

**Table 3:** Top 50 municipalities by dengue case rate.

Municipality code	Municipality	Total cases	Population size	Cases per 100 k	Hospitalizations	Hospitalizations per 100 k	Multivariate cluster
4110052	Iguatu (PR)	805	2,162	37,234	11	508.8	0
4103057	Boa Vista Da Aparecida (PR)	2,822	8,034	35,126	108	1,344.3	0
3146602	Paiva (MG)	468	1,498	31,246	0	0.0	0
3125507	São Gonçalo Do Rio Preto (MG)	964	3,098	31,117	1	32.3	0
3142502	Monjolos (MG)	681	2,196	31,011	8	364.3	0
4101002	Ampére (PR)	6,192	20,199	30,655	56	277.2	0
4113734	Luiziana (PR)	2,050	6,696	30,615	6	89.6	0
4115101	Mariluz (PR)	2,998	9,934	30,179	76	765.0	0
3113503	Carbonita (MG)	2,574	8,633	29,816	18	208.5	0
3111903	Cana Verde (MG)	1,592	5,356	29,724	1	18.7	0
4321402	Tenente Portela (RS)	4,363	14,811	29,458	171	1,154.5	0
4120358	Pranchita (PR)	1,703	5,833	29,196	17	291.4	0
4123824	Santa Lúcia (PR)	1,063	3,657	29,068	8	218.8	0
3141900	Minduri (MG)	1,103	3,815	28,912	3	78.6	0
3118304	Conselheiro Lafaiete (MG)	39,395	137,980	28,551	539	390.6	0
3168804	Tiradentes (MG)	2,275	8,008	28,409	8	99.9	0
3103801	Arapuá (MG)	743	2,674	27,786	12	448.8	0
4126678	Tamarana (PR)	2,948	10,645	27,694	57	535.5	0
4110805	Iretama (PR)	2,957	10,843	27,271	52	479.6	0
4106571	Cruzeiro Do Iguaçu (PR)	1,136	4,171	27,236	13	311.7	0
4120655	Quarto Centenário (PR)	1,069	4,170	25,636	14	335.7	0
3120839	Cuparaque (MG)	1,020	3,994	25,538	6	150.2	0
5207105	Diorama (GO)	516	2,023	25,507	57	2,817.6	0
3114303	Carmo Do Paranaíba (MG)	7,572	29,899	25,325	373	1,247.5	0
4121109	Quinta Do Sol (PR)	1,279	5,060	25,277	84	1,660.1	0
4116604	Nova América da Colina (PR)	833	3,299	25,250	2	60.6	0
3115359	Catas Altas (MG)	1,424	5,668	25,124	2	35.3	8
4113403	Leópolis (PR)	939	3,751	25,033	7	186.6	0
4116950	Nova Esperança Do Sudoeste (PR)	1,430	5,744	24,896	29	504.9	0
3109006	Brumadinho (MG)	10,119	40,777	24,816	160	392.4	0
4302378	Bom Progresso (RS)	528	2,134	24,742	0	0.0	0
3135704	Jequitibá (MG)	1,501	6,098	24,615	5	82.0	8
3131703	Itabira (MG)	28,964	117,747	24,599	738	626.8	0
3149606	Pequi (MG)	1,042	4,258	24,472	4	93.9	8
4127858	Três Barras Do Paraná (PR)	2,710	11,197	24,203	7	62.5	0
4119004	Pérola D’Oeste (PR)	1,501	6,235	24,074	39	625.5	0
3161205	São Francisco De Paula (MG)	1,483	6,293	23,566	6	95.3	8
3170701	Varginha (MG)	33,486	142,802	23,449	517	362.0	0
3149200	Pedrinópolis (MG)	798	3,404	23,443	1	29.4	0
4305207	Cerro Largo (RS)	3,254	14,009	23,228	248	1,770.3	0
4124020	Santa Tereza Do Oeste (PR)	3,193	13,749	23,224	20	145.5	0
4112207	Janiópolis (PR)	1,354	5,835	23,205	17	291.3	13
4315057	Porto Mauá (RS)	502	2,176	23,070	16	735.3	0
3168309	Taquaraçu De Minas (MG)	1,001	4,368	22,917	13	297.6	0
4122503	Roncador (PR)	2,592	11,371	22,795	3	26.4	0
4109609	Guaratuba (PR)	10,074	44,323	22,729	15	33.8	0
4312302	Miraguaí (RS)	1,024	4,506	22,725	7	155.3	0
4106308	Corbélia (PR)	4,055	17,862	22,702	55	307.9	13
4120507	Primeiro De Maio (PR)	2,287	10,121	22,597	29	286.5	13
3150505	Pimenta (MG)	1,977	8,794	22,481	30	341.1	0

Despite the strong spatial signal, a subset of municipalities with extreme case rates remained unclustered because no similar-burden neighbors existed within the DBSCAN neighborhood radius. For example, Iguatu and Boa Vista da Aparecida in Paraná, and Paiva and Monjolos in Minas Gerais each exceeded 30,000 cases per 100,000 but were algorithmically excluded as noise (Supplementary Table S3). In total, 50 municipalities surpassed 22,000 cases per 100,000, many of which appear in the ranked bar plot of the top 50 municipalities (Figure 4B; Table 3). Presenting these outliers separately ensures that single-municipality hotspots are not eclipsed by density-based methods.

Monthly DBSCAN runs revealed stability in the overall footprint of clustering across the year. The majority of municipalities were consistently assigned to clusters, and large proportions of the national population resided in municipalities persisting in clusters for ≥3 consecutive months (Supplementary Table S4). Small-multiple maps (Supplementary Figure S2) highlight waxing and waning seasonal dynamics, yet the reappearance of the same macro-areas across months suggests structural vulnerability layered on seasonal forcing.

At national scale, monthly total dengue cases rose sharply early in 2024, aligning the seasonal maximum of CHIRPS precipitation (Figure 5) (37, 38). Macroregional correlation analyses confirmed positive rainfall-dengue associations, with Spearman coefficients strenthening from lag 0 to lag 2-3 months (Supplementary Table S5), consistent with prior studies (29–31). The strongest associations were observed in the Southeast and Central-West, while the South displayed positive but more modest correlations, patterns consistent with *Aedes aegypti* biology (10–13). A case study from Jequitibá (Minas Gerais, Cluster 8) reinforced this relationship where case surges in February–March closely followed local peak precipitation, and mean annual temperatures remained within the optimal range for vectorial capacity (23–26 °C).

Socioeconomic comparisons revealed that clustered municipalities had lower GDP per capita than non-clustered ones. Among the 5,519 municipalities, those in clusters had a lower average (26,891 BRL) and median (19,367 BRL) GDP per capita compared to non-clustered municipalities (33,743 BRL and 28,687 BRL, respectively). Logistic regression confirmed a modest but statistically significant inverse association between GDP per capita and the likelihood of cluster membership (OR ≈ 0.999994, *p* < 0.001). While the effect size is small, the direction of association aligns with literature linking poverty, inadequate water infrastructure, and limited health system access to arboviral vulnerability (15–18).

## Discussion

We present a national application of multivariate DBSCAN that integrates municipal geography with epidemiological burden to delineate dengue hotspots in Brazil’s record 2024 epidemic year. This approach offers three operational benefits. First, it identifies compact municipal clusters of exceptionally high cases and hospitalization rates, which represent natural focal areas for targeted vector control and surge capacity planning. Second, it preserves algorithmic “noise,” ensuring that municipalities with extreme but isolated burdens, often overlooked in density-based clustering, remain visible and actionable. Third, monthly repetitions of the analysis reveal temporal stability, highlighting persistent hot zones where interventions should be sustained across consecutive months. The month-to-month persistence of the same municipal hotspots, despite seasonal waxing and waning, signals structural vulnerability, arguing for sustained, area-based vector control and pre-positioned clinical surge capacity rather than episodic, reactive campaigns.

The clustering patterns we identified, particularly in Minas Gerais, Paraná, and Bahia, are parts of the South and Central-West, mirror the strong autocorrelation measured Moran’s I and are consistent with known ecological and infrastructural dengue drivers (4, 10, 15, 16, 19). The rainfall-dengue correlations reinforce the established expectation of positive associations at one- to three-month lags, consistent with *Aedes aegypti* life cycles and with previous findings in Brazil and elsewhere (27, 28). Case studies like Jequitibá highlight the close coupling of rainfall and dengue incidence at the municipal level, underlining the importance of integrating environmental data into early warning systems.

Methodologically, DBSCAN demonstrated several strengths compared to traditional approaches. Unlike k-means or hierarchical clustering, DBSCAN does not assume spherical clusters or pre-specify cluster number, enabling detection of irregularly shaped and context-specific hotspots (6, 7). Its explicit handling of “noise” is particularly valuable in national applications, where isolated municipalities may experience extreme outbreaks despite lacking nearby peers.

Our sensitivity analyses confirmed overall cluster geography was robust across parameter ranges (Supplementary Table S1). However, DBSCAN’s reliance on density continuity limits its ability to capture isolated hotspots, underscoring the need for complementary approaches such as Getis-Ord Gi* or Kulldorff’s scan statistics to ensure comprehensive hotspot detection.

Importantly, incorporating hospitalization rates into the clustering process allowed us to capture both transmission intensity and disease severity. Clusters in Paraná and southern Brazil reached hospitalization rates exceeding 200–600 per 100,000, highlighting areas of potential health system overload. These findings resonate with reports of higher severity among older populations and those with comorbidities, particularly during the southern epidemic wave (3, 4).

We acknowledge that case notifications in SINAN, particularly during epidemic peaks, are not often accompanied by systematic laboratory confirmation. Nonetheless, these clinically reported cases represent the operational data stream available for epidemic management in Brazil and are the same signals upon which national response planning relies. By demonstrating that robust spatial clusters emerge even under these constraints, our analysis underscores the utility of geospatial clustering as a pragmatic surveillance tool that complements but does not replace virologic confirmation. Moreover, the congruence of our results with known ecological drivers and hospitalization patterns affirms that signal-to-noise-ratios in the surveillance system are sufficient to identify meaningful hotspots.

The socioeconomic analyses further underscore the role of structural vulnerability in shaping dengue risk. Clustered municipalities tended to be less economically advantaged, with lower GDP per capita. While the observed effect sizes were modest, this directionality aligns with evidence that poverty, water storage practices, sanitation gaps, and housing conditions amplify arboviral exposure (15–18, 23). Future studies should integrate richer structural indicators, such as sanitation coverage, water intermittency, urban density, and health systems access, to evaluate multivariable predictors of cluster membership and persistence.

From a policy perspective, our findings emphasize that dengue control in Brazil cannot rely on aggregate national metrics alone. The identification of small, localized but high-burden clusters highlight the need for municipal and regional-level targeting of vector control, diagnostic distribution, and hospital surge planning. Moreover, the observed rainfall-dengue lagged correlations support the integration of climate data into predictive modeling and early warning systems, an especially urgent need as climate variability increases (20–22, 24).

Finally, DBSCAN remains underutilized in Latin American public health surveillance despite its adaptability, scalability, and compatibility with open-source workflows. Prior studies in Southeast Asia and the Caribbean have applied DBSCAN successfully to arboviral clustering (8, 9), but national applications in Brazil remain rare. Our analysis demonstrates its feasibility and value at the municipal scale, offering a flexible geospatial tool that can complement existing surveillance systems.

Looking forward, DBSCAN-based clustering, paired with Earth observation, climate predictors, and sociodemographic indicators, can underpin predictive analytics and decentralized epidemic intelligence, improving equity through more precise, and timely interventions.

## Limitations

Several limitations should be noted. First, although DBSCAN effectively delineates coherent high-burden clusters, its reliance on local density continuity inevitably excludes single municipalities with exceptionally high rates when they lack comparable neighbors. We partially mitigate this limitation by preserving these outliers in separate panels, but complementary methods such as Kulldorff’s spatial scan or Getis-Ord Gi* could further capture such isolated hotspots. Second, our reliance on monthly CHIRPS precipitation (37, 38) improves upon single-station data by providing uniform national coverage, but may still may obscure localized microclimatic variability (28) compared with INMET station data (26). Third, the use of routine surveillance data, often unconfirmed by laboratory diagnostics, introduces potential for misclassification; however, this reflects the operational reality of epidemic response and underscores the importance of methods that can extract robust signals from imperfect data. Finally, GDP per capita is a crude proxy of socioeconomic vulnerability and should be complemented in future work with more granular indicators of water, sanitation, housing, and health system capacity. Despite these limitations, the methodological transparency, reproducibility, and national coverage of our analysis position DBSCAN clustering as a valuable addition to the toolkit for epidemic intelligence in Brazil.

## Conclusion

In Brazil’s unprecedented 2024 dengue year, multivariate DBSCAN uncovered compact municipal clusters of high burden and preserved isolated outliers that demand targeted action. Monthly clustering showed persistence of risk in the same macro-areas across seasons, while rainfall correlations at short lags confirmed expected climate-epidemic coupling. The method is transparent, scalable, and immediately useful for prioritizing vector control, diagnostics, and hospital surge planning at municipal scale. As Brazil advances decentralized surveillance and climate-aware preparedness, density-based geospatial clustering can help bridge the gap between national statistics and neighborhood-level action.

## Data Availability

The original contributions presented in the study are included in the article/Supplementary material, further inquiries can be directed to the corresponding author.

## References

[1] Gubler DJ. Epidemic dengue/dengue hemorrhagic fever as a public health, social and economic problem in the 21st century. Trends Microbiol. (2002) 10:100–3. doi: 10.1016/s0966-842x(01)02288-0 11827812

[2] Teixeira MG Costa MC Coelho G Barreto ML. Dengue: twenty-five years since reemergence in Brazil. Cad Saude Publica. (2013) 29:7–18. doi: 10.1590/S0102-311X2013000100002 19287868

[3] Figueiredo L Rocha D Costa M Pellini ACG Felix AC Luna E. A spatial case–control study on symptomatic and inapparent dengue infections in an endemic city in Brazil. Rev Inst Med Trop Sao Paulo. (2024) 66:e12. doi: 10.1590/s1678-9946202466012, PMID: 38381897 PMC10881063

[4] Souza CDF Nascimento RPS Bezerra-Santos M Armstrong ADC Gomes OV Nicácio JM. Space-time dynamics of the dengue epidemic in Brazil, 2024: an insight for decision making. BMC Infect Dis. (2024) 24:1056. doi: 10.1186/s12879-024-09813-z 39333905 PMC11430439

[5] Ministério da Saúde (BR). Sistema de Informação de Agravos de Notificação (SINAN). Dengue case notifications, 2007–2024. (2025). Available online at: https://datasus.saude.gov.br/ (Accessed June, 2025).

[6] Ester M Kriegel H-P Sander J Xu X. (1996). “A density-based algorithm for discovering clusters in large spatial databases with noise.” in *Proc. 2nd int. conf. Knowledge discovery and data mining (KDD ‘96)*. pp. 226–231.

[7] Schubert E Sander J Ester M Kriegel H-P Xu X. DBSCAN revisited, revisited: why and how you should (still) use DBSCAN. ACM Trans Database Syst. (2017) 42:21. doi: 10.1145/3068335

[8] Chen Y Liu H. Analyzing spatial patterns of dengue cases using DBSCAN clustering in Kaohsiung, Taiwan. Appl Geogr. (2018) 92:20–8. doi: 10.1016/j.apgeog.2018.02.005

[9] Zhao Y Kong Q Yu J. Detecting clusters of infectious diseases using spatial DBSCAN and surveillance data. Int J Environ Res Public Health. (2021) 18:6397. doi: 10.3390/ijerph18126434199174

[10] Barcellos C Matos V Lana RM Lowe R. Climate change, thermal anomalies, and the recent progression of dengue in Brazil. Sci Rep. (2024) 14:5948. doi: 10.1038/s41598-024-58202-8 38467690 PMC10928122

[11] Ahmed W Li C Lin Y Wang H. Modified space–time DBSCAN for spatiotemporal clustering in infectious-disease monitoring. Remote Sens. (2023) 15:88. doi: 10.3390/rs15010088

[12] Pirani M Lorenz C de Azevedo TS Barbosa GL Blangiardo M Chiaravalloti-Neto F. Effects of the El Niño-Southern Oscillation and seasonal weather conditions on Aedes aegypti infestation in the State of São Paulo (Brazil): A Bayesian spatio-temporal study. PLoS Negl Trop Dis. (2024) 18:e0012397. doi: 10.1371/journal.pntd.0012397 39264869 PMC11392405

[13] Santos CAG Guerra-Gomes IC Gois BM Peixoto RF Keesen TSL da Silva RM. Correlation of dengue incidence and rainfall occurrence using wavelet transform for João Pessoa city. Sci Total Environ. (2019) 647:794–805. doi: 10.1016/j.scitotenv.2018.08.019 30096669

[14] Instituto Brasileiro de Geografia e Estatística (IBGE). Population and GDP by municipality. (2025). Available online at: https://www.ibge.gov.br/estatisticas (Accessed June, 2025)

[15] Lee SA Economou T de Castro Catão R Barcellos C Lowe R. The impact of climate suitability, urbanisation, and connectivity on the expansion of dengue in 21st century Brazil. PLoS Negl Trop Dis. (2021) 54:e0009773. doi: 10.1371/journal.pntd.0009773 PMC8691609 34882679

[16] Caprara A Lima JW Marinho AC Calvasina PG Landim LP Sommerfeld J. Irregular water supply, household usage and dengue: a bio-social study in the Brazilian northeast. Cad Saude Publica. (2009) 25:S125–36. doi: 10.1590/s0102-311x2009001300012 19287857

[17] Whiteman A Gómez MM Tsetsarkin K Poh KC Watkins AS Lucas KJ. Do socioeconomic factors drive *Aedes* mosquito vectors and their arboviral diseases? A systematic review of dengue, chikungunya, yellow fever, and Zika Virus. One Health. (2020) 11:100188. doi: 10.1016/j.onehlt.2020.100188, PMID: 33392378 PMC7772681

[18] Queiroz L Medronho RA. Spatial analysis of the incidence of dengue, zika and chikungunya and socioeconomic determinants in the city of Rio de Janeiro, Brazil. Epidemiol Infect. (2021) 149:e188. doi: 10.1017/S0950268821001882 34338179 PMC8365848

[19] Castro MC Wilson ME Bloom DE. Disease and economic burdens of dengue. Lancet Infect Dis. (2017) 17:e70–8. doi: 10.1016/S1473-3099(16)30545-X 28185869

[20] Silva FD dos Santos AM Corrêa Rda G Caldas Ade J. Temporal relationship between rainfall, temperature and dengue in São Luís, Maranhão. Ciênc Saúde Colet. (2016) 21:641–6. doi: 10.1590/1413-81232015212.09592015 26910171

[21] Oliveira Roster K Martinelli T Connaughton C Santillana M Rodrigues FA. Impact of the COVID-19 pandemic on dengue in Brazil: Interrupted time series analysis of changes in surveillance and transmission. PLoS Negl Trop Dis. (2024) 18:e0012726. doi: 10.1371/journal.pntd.0012726 39724056 PMC11709241

[22] Chen X Moraga P. Assessing dengue forecasting methods: a comparative study of statistical models and machine learning in Rio de Janeiro. Trop Med Health. (2025) 53:52. doi: 10.1186/s41182-025-0052-7 40211309 PMC11984044

[23] Lowe R Lee SA O'Reilly KM Brady OJ Bastos L Carrasco-Escobar G. Combined effects of hydrometeorological hazards and urbanisation on dengue risk in Brazil: a spatiotemporal modelling study. Lancet Planet Health. (2021) 5:e209–19. doi: 10.1016/S2542-5196(20)30292-8, PMID: 33838736

[24] Daudt-Lemos M Ramos-Silva A Faustino R Noronha TG Vianna RAO Cabral-Castro MJ. Rising incidence and spatiotemporal dynamics of emerging and reemerging arboviruses in Brazil. Viruses. (2025) 17:158. doi: 10.3390/v17020158, PMID: 40006913 PMC11860164

[25] de Castro-Nunes P Palmieri P Simões PP Rodrigues de Carvalho PV Jatobá A. Leveraging machine learning on hospitalizations in the dynamics of dengue spread in Brazil: an ecological study of health systems resilience. Lancet Reg Health Am. (2025) 44:101042. doi: 10.1016/j.lana.2025.101042 40065770 PMC11891147

[26] Brady OJ Gething PW Bhatt S Messina JP Brownstein JS Hoen AG. Refining the global spatial limits of dengue virus transmission by evidence-based consensus. PLoS Negl Trop Dis. (2012) 6:e1760. doi: 10.1371/journal.pntd.0001760, PMID: 22880140 PMC3413714

[27] Roster K Connaughton C Rodrigues FA. Machine-learning-based forecasting of dengue fever in Brazilian cities using epidemiologic and meteorological variables. Am J Epidemiol. (2022) 191:1803–1812. doi: 10.1093/aje/kwac090 35584963

[28] Stolerman LM Maia PD Kutz JN. Forecasting dengue fever in Brazil: an assessment of climate conditions. PLoS One. (2019) 14:e0220106. doi: 10.1371/journal.pone.0220106, PMID: 31393908 PMC6687106

[29] Eisen L Lozano-Fuentes S. Use of mapping and spatial and space-time modeling approaches in operational control of *Aedes aegypti* and dengue. PLoS Negl Trop Dis. (2009) 3:e411. doi: 10.1371/journal.pntd.0000411, PMID: 19399163 PMC2668799

[30] Chen X Moraga P. Forecasting dengue across Brazil with LSTM neural networks. BMC Public Health. (2025) 25:973. doi: 10.1186/s12889-025-0973-0 40075398 PMC11900637

[31] Borges IVG Musah A Dutra LMM Tunali M Lima CL Tunali MM. Analysis of the interrelationship between precipitation and confirmed dengue cases in the city of Recife (Brazil) covering climate and public health information. Front Public Health. (2024) 12:1456043. doi: 10.3389/fpubh.2024.1456043, PMID: 39507663 PMC11537940

[32] Messina JP Brady OJ Golding N Kraemer MUG Wint GRW Ray SE. The current and future global distribution and population at risk of dengue. Nat Microbiol. (2019) 4:1508–15. doi: 10.1038/s41564-019-0476-8, PMID: 31182801 PMC6784886

[33] Liu-Helmersson J Brännström Å Sewe MO Semenza JC Rocklöv J. Estimating past, present, and future trends in the global distribution and abundance of *Aedes aegypti* mosquitoes. PLoS One. (2014) 9:e89783. doi: 10.1371/journal.pone.0089783, PMID: 31249824 PMC6582658

[34] Instituto Nacional de Meteorologia (INMET). (n.d.). Meteorological time series. Available online at: https://portal.inmet.gov.br/dadoshistoricos (Accessed June, 2025)

[35] Lowe R Barcellos C Brasil P Cruz OG Honório NA Kuper H. The zika virus epidemic in Brazil: from discovery to future implications. Int J Environ Res Public Health. (2017) 14:9. doi: 10.3390/ijerph14010009 PMC5800195 29315224

[36] Bhatt S Gething PW Brady OJ Messina JP Farlow AW Moyes CL. The global distribution and burden of dengue. Nature. (2013) 496:504–7. doi: 10.1038/nature12060, PMID: 23563266 PMC3651993

[37] Funk C Peterson P Landsfeld M Pedreros D Verdin J Shukla S. The climate hazards infrared precipitation with stations--a new environmental record for monitoring extremes. Sci Data. (2015) 2:150066. doi: 10.1038/sdata.2015.66 26646728 PMC4672685

[38] Climate Hazards Center. Climate Hazards Group InfraRed Precipitation with Station data (CHIRPS). University of California, Santa Barbara. Available online at: https://www.chc.ucsb.edu/data/chirps(Accessed June 2025).

