# Supplementary material for: Geospatial clustering reveals dengue hotspots across Brazilian municipalities, 2024
Source: Front Public Health. 2025 Oct 27;13:1620914. doi: 10.3389/fpubh.2025.1620914 (PMC12597951; doi:10.3389/fpubh.2025.1620914)
Supplement: Supplementary file 1 [file Table_1.docx]

**Supplementary Table S1**. DBSCAN Sensitivity Analysis (eps and minPts)

| **Parameter Setting** | **Number of Noise Points** | **Number of Clusters** |
| --- | --- | --- |
| eps = 0.2, minPts = 3 | 1,049 | 71 |
| eps = 0.2, minPts = 5 | 1,390 | 32 |
| eps = 0.3, minPts = 3 | 408 | 25 |
| eps = 0.3, minPts = 5 | 557 | 17 |
| eps = 0.4, minPts = 3 | 222 | 15 |
| eps = 0.4, minPts = 5 | 306 | 1 |
