# Supplementary material for: Geospatial clustering reveals dengue hotspots across Brazilian municipalities, 2024
Source: Front Public Health. 2025 Oct 27;13:1620914. doi: 10.3389/fpubh.2025.1620914 (PMC12597951; doi:10.3389/fpubh.2025.1620914)
Supplement: Supplementary file 2 [file Table_2.docx]

**Supplementary Table S2.** Global Moran’s I Test Results for Spatial Autocorrelation of Dengue Case Rates

| **Statistic** | **Value** |
| --- | --- |
| Moran’s I statistic | 0.598 |
| Expected value under null | -0.00018 |
| Variance | 6.74 x 10-5 |
| Standard deviate (z-score) | 72.797 |
| p-value | <0.001 |
