# Supplementary material for: Geospatial clustering reveals dengue hotspots across Brazilian municipalities, 2024
Source: Front Public Health. 2025 Oct 27;13:1620914. doi: 10.3389/fpubh.2025.1620914 (PMC12597951; doi:10.3389/fpubh.2025.1620914)
Supplement: Supplementary file 3 [file Table_3.docx]

**Supplementary Table S3**. Outlier Municipalities with Case Rates >30,000 per 100k, Unclustered

| **Municipality Code** | **Municipality Name** | **State** | **Cases per 100k** | **Hospitalizations per 100k** |
| --- | --- | --- | --- | --- |
| **3125507** | São Gonçalo do Rio Preto | MG | 31,117 | 32.3 |
| **3142502** | Monjolos | MG | 31,011 | 364.3 |
| **3146602** | Paiva | MG | 31,242 | 0 |
| **4101002** | Ampére | PR | 30,655 | 277.2 |
| **4103057** | Boa Vista da Aparecida | PR | 35,126 | 1,344.3 |
| **4110052** | Iguatu | PR | 37,234 | 508.8 |
| **4113734** | Luiziana | PR | 30,615 | 89.6 |
| **4115101** | Mariluz | PR | 30,179 | 765.0 |

Municipalities with dengue case rates exceeding 30,000 per 100,000 inhabitants but not assigned to a DBSCAN cluster. These isolated hotspots highlight the need for local interventions even in the absence of broader spatial clustering.
