# Supplementary material for: Geospatial clustering reveals dengue hotspots across Brazilian municipalities, 2024
Source: Front Public Health. 2025 Oct 27;13:1620914. doi: 10.3389/fpubh.2025.1620914 (PMC12597951; doi:10.3389/fpubh.2025.1620914)
Supplement: Supplementary file 4 [file Table_4.docx]

**Supplementary Table S4: Monthly DBSCAN Cluster Summary**

| Month | Number of clusters | Municipalities clustered (%) | Population in persistent clusters (>=3 mo, thousands) |
| --- | --- | --- | --- |
| 1 | 8 | 98.2% | 49,671 |
| 2 | 9 | 98.6% | 49,855 |
| 3 | 7 | 98.6% | 49,926 |
| 4 | 7 | 98.8% | 49,997 |
| 5 | 8 | 98.9% | 50,034 |
| 6 | 9 | 98.6% | 49,957 |
| 7 | 8 | 97.7% | 49,776 |
| 8 | 11 | 97.9% | 49,814 |
| 9 | 5 | 98.1% | 49,839 |
| 10 | 10 | 98.3% | 49,894 |
| 11 | 7 | 98.5% | 49,900 |
| 12 | 12 | 98.7% | 49,936 |
