# Supplementary material for: Geospatial clustering reveals dengue hotspots across Brazilian municipalities, 2024
Source: Front Public Health. 2025 Oct 27;13:1620914. doi: 10.3389/fpubh.2025.1620914 (PMC12597951; doi:10.3389/fpubh.2025.1620914)
Supplement: Supplementary file 5 [file Table_5.docx]

**Supplementary Table S5: Spearman correlations (r) between precipitation and dengue case rates by region and lag (2024)**

N = number of municipality-month observations. Lag = months between precipitation and dengue cases*.*

| Region | Lag (months) | Spearman r | p-value | N* |
| --- | --- | --- | --- | --- |
| Central-West | 0 | 0.062 | 4.14 x 10^-6^ | 5,568 |
|  | 1 | 0.331 | 8.4 x 10^-131^ | 5,104 |
|  | 2 | 0.501 | 4.2 x 10^-294^ | 4,640 |
|  | 3 | 0.493 | 1.2 x 10^-254^ | 4,176 |
| Northeast | 0 | 0.268 | <1 x 10^-321^ | 19,786 |
|  | 1 | 0.360 | <1 x 10^-322^ | 18,137 |
|  | 2 | 0.346 | <1 x 10^-322^ | 16,488 |
|  | 3 | 0.264 | 3.0 x 10^-234^ | 14,839 |
| North | 0 | 0.294 | 1.5 x 10^-93^ | 4,651 |
|  | 1 | 0.394 | 4.0 x 10^-158^ | 4,260 |
|  | 2 | 0.385 | 9.2 x 10^-137^ | 3,869 |
|  | 3 | 0.312 | 1.4 x 10^-79^ | 3,478 |
| Southeast | 0 | 0.065 | 1.5 x 10^-19^ | 19,074 |
|  | 1 | 0.354 | <1 x 10^-322^ | 17,484 |
|  | 2 | 0.509 | <1 x 10^-322^ | 15,894 |
|  | 3 | 0.501 | <1 x 10^-322^ | 14,304 |
| South | 0 | 0.027 | 0.0016 | 13,320 |
|  | 1 | 0.095 | 4.1 x 10^-26^ | 12,210 |
|  | 2 | 0.073 | 1.8 x 10^-14^ | 11,100 |
|  | 3 | 0.046 | 5.30 x 10^-6^ | 9,990 |
