# Supplementary figures and images for: Geospatial clustering reveals dengue hotspots across Brazilian municipalities, 2024
Source: Front Public Health. 2025 Oct 27;13:1620914. doi: 10.3389/fpubh.2025.1620914 (PMC12597951; doi:10.3389/fpubh.2025.1620914)

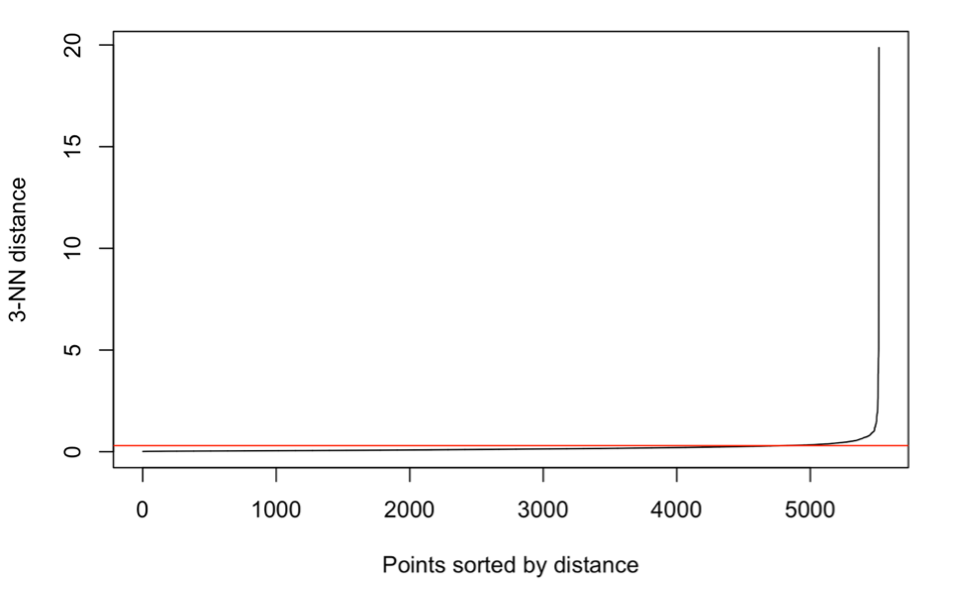

Supplement: Supplementary file 6 [file Image_1.png]

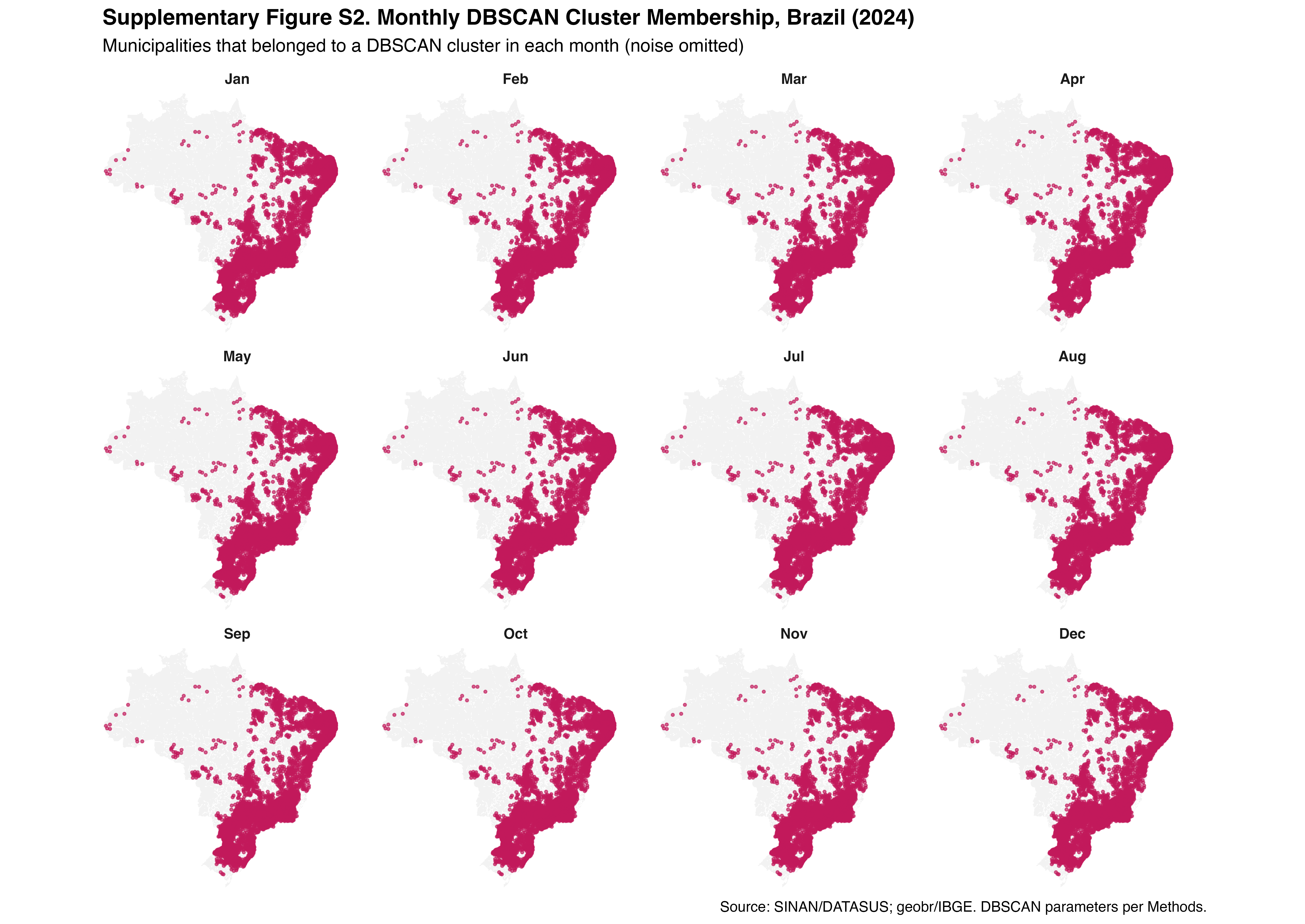

Supplement: Supplementary file 7 [file Image_2.png]

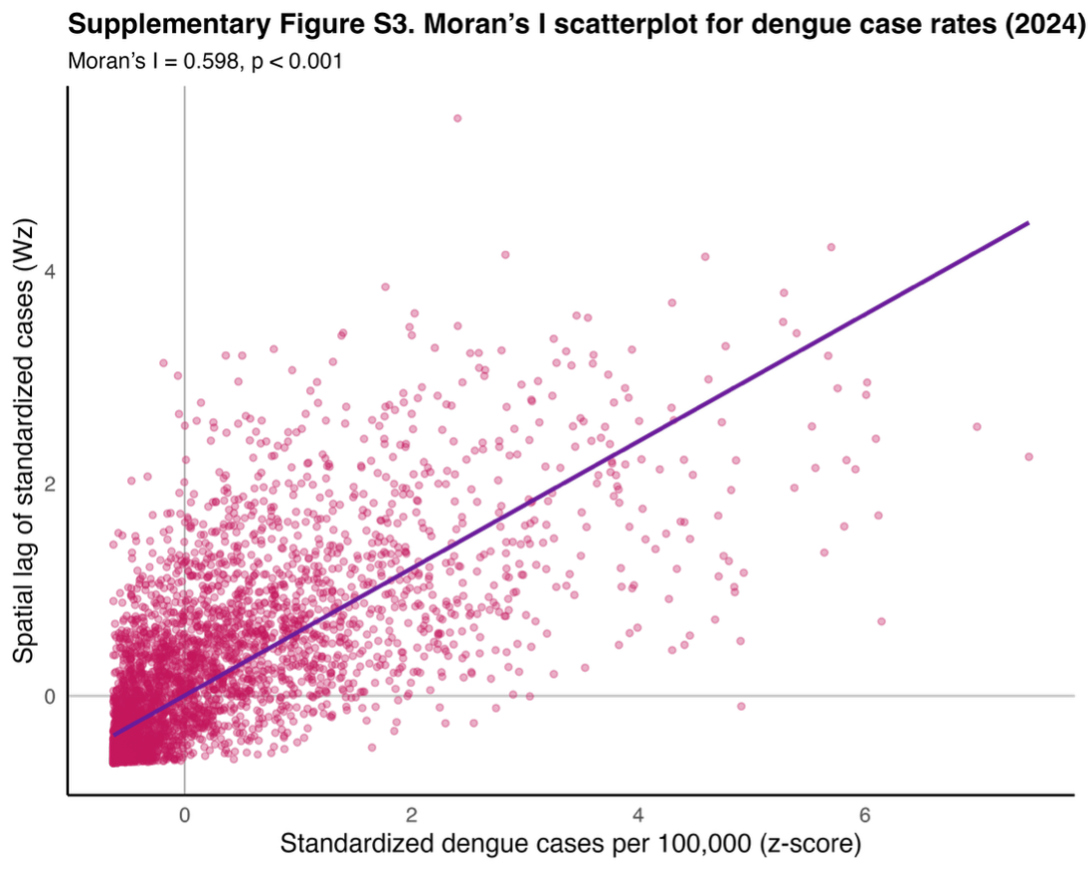

Supplement: Supplementary file 8 [file Image_3.png]

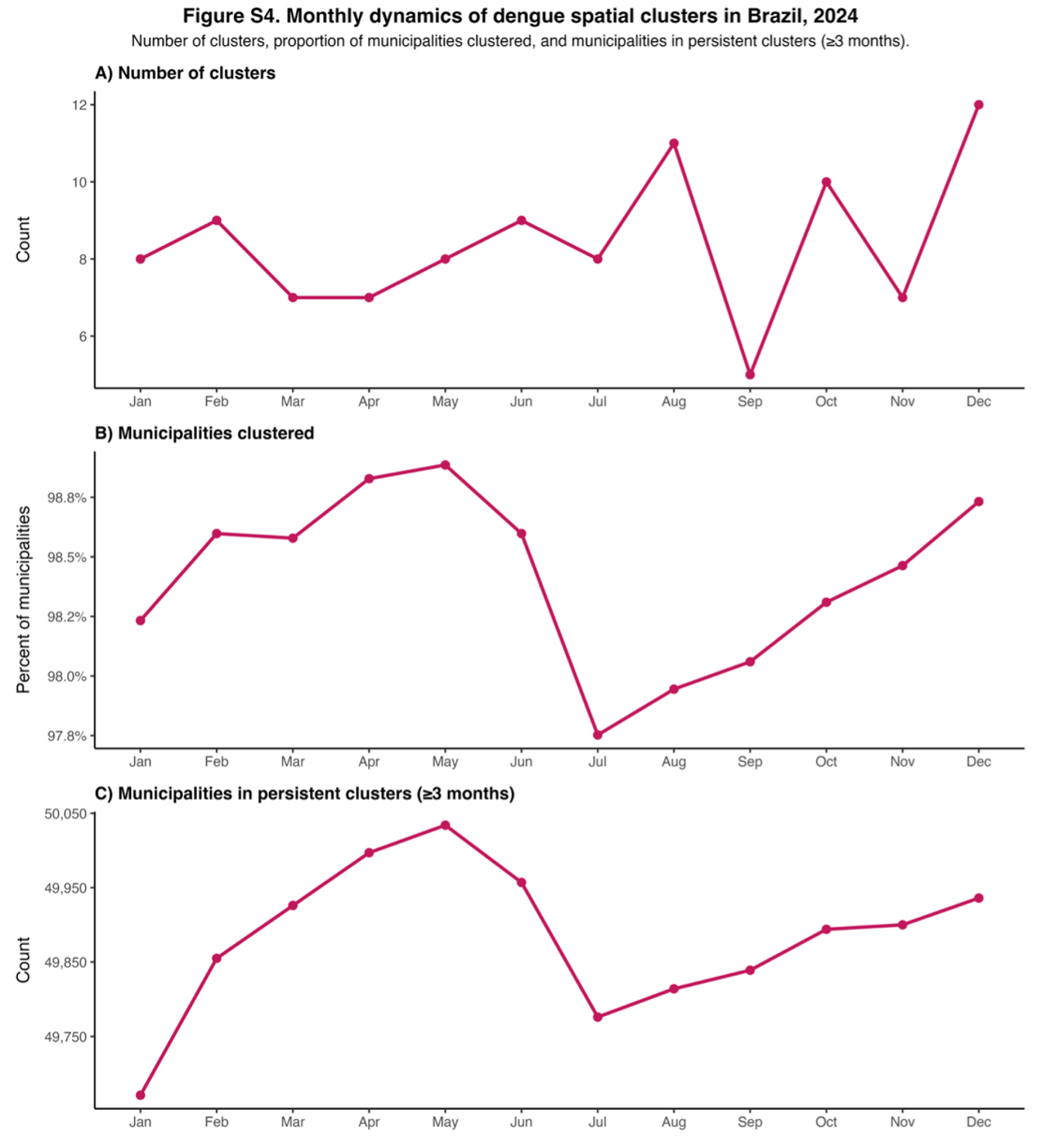

Supplement: Supplementary file 9 [file Image_4.png]
